# Supplementary material for: The interactome of intact mitochondria by cross-linking mass spectrometry provides evidence for coexisting respiratory supercomplexes
Source: Mol Cell Proteomics. 2017 Dec 8;17(2):216–32. doi: 10.1074/mcp.RA117.000470 (PMC5795388; doi:10.1074/mcp.RA117.000470)
Supplement: Supplemental Data [file supp_RA117.000470_133922_0_supp_23978_fzffwf.zip › spectra_annotation/mito_DR_spectra_annotation/16-1-12-1-4-1.pdf]

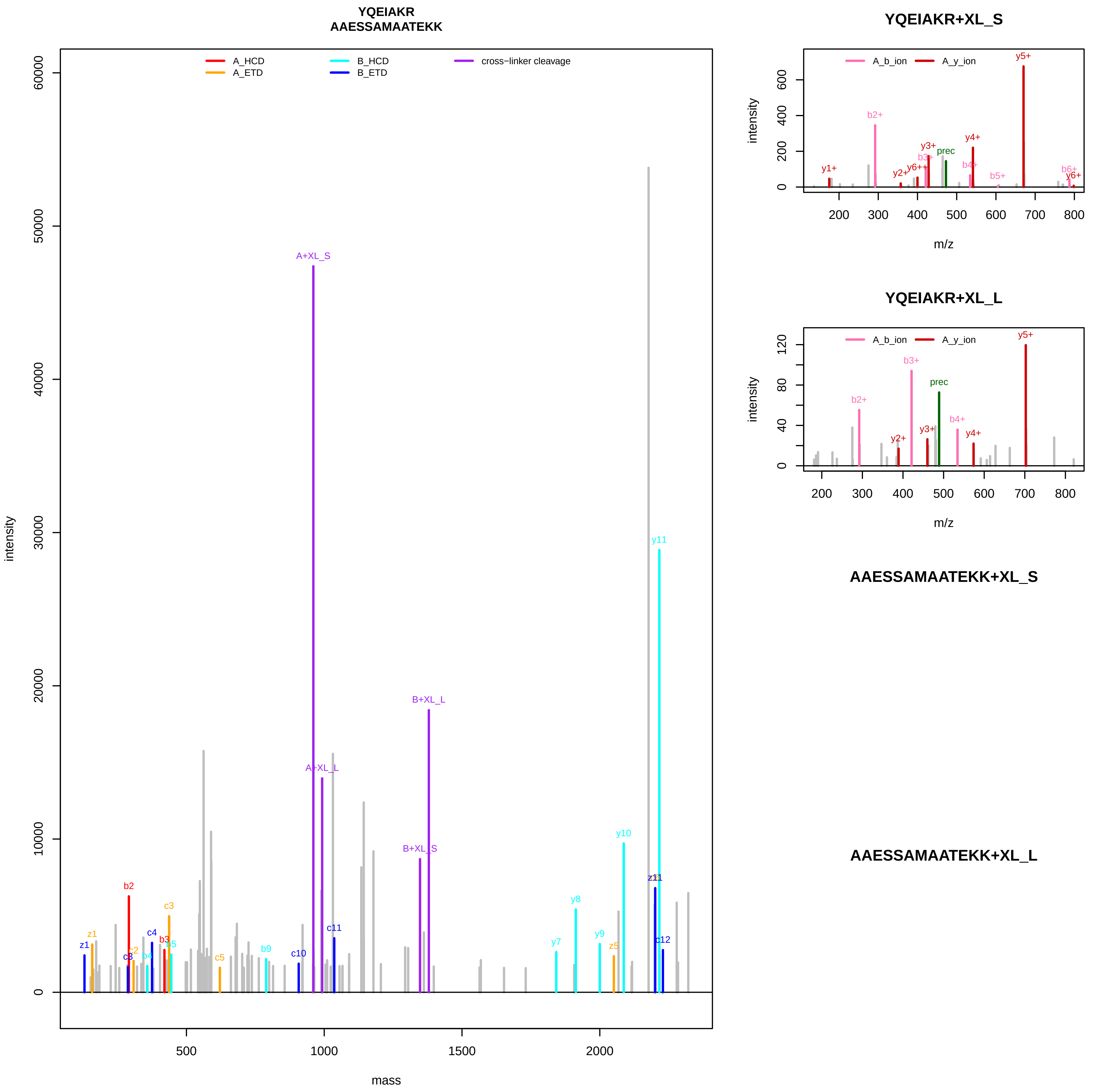
YQEIAKR
AAESSAMAATEKK
A_HCD
A_ETD
B_HCD
B_ETD
cross−linker cleavage
A+XL_S
B+XL_L
A+XL_L
B+XL_S
y11
y10
y8
y7
y9
z5
z11
c12
c11
c10
b9
c5
c3
b2
b3
b5
b4
c4
c3
c2
z1
z1
intensity
mass
YQEIAKR+XL_S
A_b_ion
A_y_ion
y5+
b2+
y4+
y3+
prec
b3+
y6++
y2+
y1+
b4+
b5+
b6+
y6+
m/z
YQEIAKR+XL_L
b3+
prec
b2+
b4+
y3+
y2+
y4+
AAESSAMAATEKK+XL_S
AAESSAMAATEKK+XL_L
